# Challenges in implementing The Institute of Medicine systematic review standards

**DOI:** 10.1186/2046-4053-2-69

**Published:** 2013-08-27

**Authors:** Stephanie M Chang, Eric B Bass, Nancy Berkman, Timothy S Carey, Robert L Kane, Joseph Lau, Sara Ratichek

**Affiliations:** 1 Agency for Healthcare Research and Quality, 540 Gaither Road, Rockville, MD 20850, USA; 2 Johns Hopkins University, Hampton House 680A, 624 N. Broadway, Baltimore, MD 21205, USA; 3 RTI International, 3040 East Cornwallis Road, Post Office Box 12194, Research Triangle Park, NC 27709-2194, USA; 4 University of North Carolina at Chapel Hill, 5039 Old Clinic Building, CB #7110, Chapel Hill, NC 27599-7110, USA; 5 University of Minnesota School of Public Health, MMC 179, Minneapolis, MN 55455, USA; 6 Brown University, 121 S. Main Street, 8th floor, Providence, RI 02912, USA; 7 University of Utah, School of Nursing, Salt Lake City, USA

**Keywords:** Systematic review methods, Institute of Medicine Standards, Evidence-based practice centers

## Abstract

In 2011, The Institute of Medicine (IOM) identified a set of methodological standards to improve the validity, trustworthiness, and usefulness of systematic reviews. These standards, based on a mix of theoretical principles, empiric evidence, and commonly considered best practices, set a high bar for authors of systematic reviews.

Based on over 15 years of experience conducting systematic reviews, the Agency for Healthcare Research and Quality Evidence-based Practice Center (EPC) program has examined the EPC’s adherence and agreement with the IOM standards. Even such a large program, with infrastructure and resource support, found challenges in implementing all of the IOM standards. We summarize some of the challenges in implementing the IOM standards as a whole and suggest some considerations for individual or smaller research groups needing to prioritize which standards to adhere to, yet still achieve the highest quality and utility possible for their systematic reviews.

## Background

As enthusiasm for evidence-based practice grows, so too have considerations of methodological standards for the underpinning systematic reviews (SRs) of the literature. These standards specify criteria to ensure that SRs employ a rigorous systematic approach and are authored by objective researchers. To be trustworthy, SRs must be transparent about the underlying logic defining the scope of the review, analysis methods synthesizing included studies, and where any elements of judgment reside. SRs must also be relevant and usable; they must address salient topics that capture new developments in a field and describe their findings clearly and concisely.

The Institute of Medicine (IOM) has laid out a set of methodological standards for SRs that adhere to the principles noted above [1]. In response to a congressional mandate for patient-centered evidence-based information, the IOM’s primary audience for these standards was public sponsors of SRs. However, these recommended standards will influence all who are involved in conducting SRs.

Over the past 15 years, the Agency for Healthcare Research and Quality Evidence-based Practice Center (EPC) Program has developed and refined explicit methods for conducting SRs [2]; this body of methodological guidance helped inform the IOM standards. The EPC Program has developed resources and infrastructure that enhance its ability to conduct SRs that adhere to rigorous methodological standards. However, full implementation of IOM standards would stretch the resources of EPCs and so is likely to stretch the limits of resources of individuals and small groups conducting SRs. The IOM standards are helpful in laying out the highest ideals, but we believe it is important to be flexible and consider efficiency when implementing the standards. In this article, we share our perspective on challenges in implementing the IOM standards and offer suggestions for how to meet the standards in the face of limited resources.

## Main text

### Challenges in implementation

The EPC program systematically gathered feedback from each EPC director to identify particular IOM recommendations that were not standard practice across EPCs. Subsequent structured discussions among workgroups of EPC investigators identified particular challenges in implementation because of difficulties in interpretation or practical limitations [3], despite overall support for the intent and principles of the IOM recommendations.

The IOM recommendations include 84 separate standards. Some of the standards require user judgment in interpretation and implementation. For example, the IOM recommends exclusion of input from individuals whose conflict of interest or bias would diminish the credibility of the report, yet recommends inclusion of individuals on the team with pertinent clinical expertise and also encourages public comment periods that allow input from those who may have inherent biases. In identifying experts, it may be difficult to disentangle intellectual or professional bias from pertinent clinical expertise. In adjudicating these seemingly contradictory recommendations, the EPCs responded by improving our definition of non-financial conflicts of interest to assess intellectual or professional biases, developing a set of questions to help project leads and funders assess for non-financial conflicts of interest [4] and revising our policy for handling conflicts. We maintain a tiered approach for allowable conflicts of interest depending on the level of involvement with the SR and ability to manage those conflicts. While we welcome and encourage public comment, we also specifically solicit input from experts without bias or with manageable biases. While the EPC approach may differ from what was envisioned by the IOM, we believe it still adheres to the principles and intent of the IOM standards.

Some of the IOM recommendations encourage a laudable level of rigor, but at a high cost. For example, the IOM recommended a number of actions that, while requiring a modest increase in cost, are likely to add significantly to the duration of the review, such as conducting an independent peer review of search strategies by a librarian, conducting a “web search”, and posting protocols for public comment. Other recommendations, such as full, duplicate independent reviews at each step of the review and assessing the strength of evidence of all outcomes, would significantly add to the personnel costs involved in the review. Although end users of research care about rigorous methodology, they also value timeliness and efficient delivery of findings. Requiring adherence to all of the standards as a whole would likely increase both the cost and duration of reviews, restricting the organizations able to conduct them to those with significant resources.

## Discussion

### A reasoned approach in the face of limited resources

We believe that systematic reviewers with limited resources will have to choose between not conducting a review and prioritizing among recommended standards. The IOM standards have not been tested, and the balance of the costs and benefits of adopting all of the standards as a whole is yet unknown. Thus, we suggest that, if required, reviewers prioritize among standards based on available evidence, anticipated cost-effectiveness, and the theoretical principles underpinning each standard, especially for those that are highly resource intensive.

Although the IOM committee based each of the standards on a combination of narrative review of the empirical evidence and expert opinion, individual organizations may choose to require a higher threshold of evidence prior to adopting that standard. For example, although a web search of the literature may capture additional studies, evidence is not conclusive that it is likely to ultimately change the findings of the review.

Reviewers must be sensitive to the danger of achieving only small, if any, marginal gains from substantial increased effort and cost. After exploring all possible options to minimize the burden (i.e., using crowd-sourced tools such as the Systematic Review Data Repository™ [5] or other software tools), reviewers may choose to prioritize among or focus the implementation of resource-intensive standards. For example, although duplicate independent actions at each step of the review process may avoid some errors, reviewers may choose to focus on the most important data items that directly affect the report findings (i.e., those used in meta-analysis).

Another approach to prioritization is to consider the underlying principle each standard is intended to address and to rank their importance (see the List of foundational principles for IOM standards). Reviewers should explicate their own values as well as those of their partner constituencies using the review. For example, a publicly funded organization may hold transparency (and thus providing opportunities for public review and comment) as a non-negotiable principle, whereas a private organization may hold efficiency and timeliness as higher values, especially if the review is for internal consumption.

### List of foundational principles for IOM standards

**Acceptability** (**credibility**)

**Applicability** (**generalizability**)

Efficiency

**Patient**-**centeredness**

Scientific rigor

Timeliness

Transparency

## Conclusions

The IOM standards were developed as the highest ideal. While we support implementation of these standards, we are concerned that the high bar may dissuade individuals and small groups with limited resources from conducting reviews. Rather than an all-or-nothing approach to rote implementation, we encourage individuals and small groups to consider the evidence and principles behind each recommendation, along with their own values and principles.

Using the IOM recommendations as the gold standard raises an ironic paradox. While intended to strengthen the science of objective empirical review, they rely primarily on best practices, theoretical benefit, and expert opinion, but as a whole are untested. SR methods are advancing rapidly, and we encourage evaluation of current practices in conducting reviews, as well as refinement of SR methods to continue to improve their validity, timeliness, and accessibility to users. We hope that the IOM contribution to this field will be considered and updated in work by other organizations as the field continues to evolve.

## Abbreviations

EPC: Evidence-based practice center; IOM: Institute of Medicine; SR: Systematic review.

## Competing interests

The authors commission (SC) or conduct (EB, NB, TC, RK, JL, SR) systematic reviews for the Agency for Healthcare Research and Quality Evidence-based Practice Center Program, but declare no other competing interests.

## Authors’ contributions

SC, NB, SR, and JL conceived of the scope of the paper. SC and SR organized discussions. RK, EB, and TC contributed to discussions further refining the paper. RK and SC participated in the initial draft with reviews and edits by all authors. All authors read and approved the final manuscript.

## Authors’ information

The EPC program is funded by the Agency for Healthcare Research and Quality to conduct systematic reviews to aid in translation of research and to promote the use of research in evidence-based decision-making.
